# Application of Industrially Produced Chitosan in the Surface Treatment of Fibre-Based Material: Effect of Drying Method and Number of Coating Layers on Mechanical and Barrier Properties

**DOI:** 10.3390/polym10111232

**Published:** 2018-11-07

**Authors:** Samir Kopacic, Andrea Walzl, Ulrich Hirn, Armin Zankel, Rudolf Kniely, Erich Leitner, Wolfgang Bauer

**Affiliations:** 1 Institute of Paper, Pulp and Fibre Technology, Graz University of Technology, Inffeldgasse 23, 8010 Graz, Austria; ulrich.hirn@tugraz.at (U.H.); rudolf.kniely@tugraz.at (R.K.); wolfgang.bauer@tugraz.at (W.B.); 2 Institute of Analytical Chemistry and Food Chemistry, Graz University of Technology, Stremayrgasse 9/2, 8010 Graz, Austria; andrea.walzl@tugraz.at (A.W.); erich.leitner@tugraz.at (E.L.); 3 Institute of Electron Microscopy and Nanoanalysis, NAWI Graz, Graz University of Technology and Centre for Electron Microscopy, Steyrergasse 17, 8010 Graz, Austria; armin.zankel@felmi-zfe.at

**Keywords:** industrially produced chitosan, surface treatment, fibre-based material, drying effects, multi-layering, barriers, mechanical properties

## Abstract

Chitosan is a versatile biopolymer with many interesting functionalities. Its effects on the barrier and mechanical properties of single- or double-coated fibre-based packaging papers in dependence on the applied drying regime were successfully tested. Our investigations revealed chitosan to be a highly robust biopolymer, since the different drying regimes did not alter its contribution to the development of strength and barrier properties of the coated packaging papers. These properties showed a stronger influence of the applied coat weights than of the different drying regimes. The effect of chitosan coatings were quantified by measuring tensile strength (TS), burst strength (BS) and tensile energy absorption (TEA). These revealed that TS, BS and TEA of the coated papers increased significantly. Moreover, the chitosan-coated papers were less permeable against water vapor and air. High grease resistance was observed for double-coated papers, irrespective of the drying regimes. The coated paper surface showed a more hydrophilic character, resulting in lower contact angles and higher water absorption properties. In this study, industrially produced chitosan has been proven to be a renewable, robust biopolymer that can be utilized as an additive to increase strength and the barrier properties of fibre-based materials.

## 1. Introduction

Sustainable and renewable materials and products are constantly gaining importance in everyday life. The extensive utilization of bio-based materials is crucial for the further development of a circular bioeconomy to decrease our dependence on petroleum-based materials. Among the many well-known applications of fibre-based materials, packaging paper and board grades for food, pharmaceuticals and cosmetics are some of the most important in our daily life [1,2,3,4,5,6]. Unlike the homogeneous synthetic polymeric material, the building units of the paper network are cellulosic fibre that are heterogeneous by nature and creating a dense structure that lacks pores or voids without additives is almost impossible. The surface of fibre-based packaging materials is porous and must be treated either by applying petroleum-based polymers, biopolymers, or blends of polymeric and inorganic minerals [7,8,9,10,11]. Because of their innate structural properties, packaging papers and boards lack crucial barrier properties against the permeation of fat, oil, water vapor or other gases. For that reason, they have to be laminated or extrusion coated with petroleum-based polymers, which ultimately affects the sustainability, recyclability and biodegradability of the final, fibre-based packaging products [12,13,14,15]. The tensile properties of paper and board can be improved by blending cellulosic fibres with functional strength additives to increase the bonding area and strength between fibres, which leads to an increase in the overall packaging strength [16,17,18,19,20].

Packaging consumption is directly related to the growth of the global population and the increase in online commerce. The results of the current forecast for the global packaging market indicate a growth in consumption of at least 4% in 2018 to a market volume of almost 1 trillion USD. The demand for functional chemicals, such as strength additives and barrier coatings for packaging applications, that today still are mainly petroleum-based, has also increased since 2015 by at least 5% per year [21,22]. Possible alternatives for the substitution of non-renewable packaging materials are combinations of paper and board with renewable biopolymers, such as industrially produced chitosan. Its production has steadily increased along with growing application in the food, textile, pharmaceutical industries, and, more recently, in the packaging industry [23,24]. Chitosan is a biopolymer which is abundant in nature, fully biodegradable and renewable and has a high potential to be used in the surface treatment of paper and board to improve its structural, mechanical and barrier properties. Chitosan is derived from chitin, found in exoskeleton of insects or crustaceans. Under defined conditions regarding pH, degree of polymerization and deacetylation, chitosan has the character of a polyelectrolyte and becomes fully water-soluble [25,26,27,28].

The chitosan as a natural material is in focus of many researchers all over the world and several references citied in our work are dealing with chitosan barrier and strength properties investigations. However, previous investigations such as influence of drying on barrier and strength properties were mostly performed using casted freestanding films. Furthermore, the cellulosic fibre based substrates that have been used for coating are laboratory-produced paper sheets, which serve as a model for possible industrial applications [29,30,31]. The results of already published work represent valuable basis for further development and practical applications of chitosan in surface treatment of fibre based packaging material presented in our work.

The subject of our investigation was an experimental study of the barrier and mechanical properties of industrial grade chitosan coated onto paper surfaces. We investigated how chitosan coated onto paper is influenced by different drying regimes used in the paper coating industry (Infrared or hot air drying). Our experimental approach evaluated whether drying could potentially influence the development of strength properties and alter the surface and barrier properties. Additionally, we evaluated the impact of two different applied coat weights by single or double coating the paper on the barrier and mechanical properties. The coating technique applied in this work was a laboratory film press, which was designed to simulate the real industrial-coating environment accurately. For the thus coated papers grease resistance, water vapor transmission rate, air permeance, wettability (contact angle) and water absorption were determined. The coated surface was visualized using scanning electron microscopy (SEM) and the most important mechanical properties of packaging materials, such as the tensile strength, burst strength, tensile energy absorption were assessed. Our results indicate that chitosan has the potential to improve both the mechanical and barrier properties of fibre-based packaging material.

## 2. Materials, Equipment and Methods

### 2.1. Raw Materials and Characterization

Industrially produced chitosan (CAS: 9012-76-4, poly-[1,4]-β-d-glucosamine) was purchased from Biolog-Heppe GmbH (Landsberg, Germany). Chitosan was supplied as a solid powder (yellow particles). The most important, product-specific data received from the chitosan producer are summarized in Table 1. The applied chitosan has a medium molecular weight and viscosity, making it suitable for application using the film press method. The substrate used for surface treatment was an industrially produced base paper from an Austrian paper mill. The base paper consists of a mix of bleached hardwood and softwood chemical pulp. The paper was mass sized using alkyl ketene dimer (AKD) and the surface was untreated.

### 2.2. Coating Machine

The laboratory coater used for surface treatment of the fibre-based substrate was a film press Coating Unit CU 5 supplied by Sumet Messtechnik GmbH (Denklingen, Germany). The coater is equipped with an applicator roll (diameter of 95 mm and width of 300 mm) and, depending on the coating material and substrate, the contact force for rolls can be adjusted (0–1150 N at 8 bar). Between the application rod and substrate, the possible contact force ranges from 0 to 230 N at 8 bar. The coater was equipped with an air-drying system (max. 2000 W) and an infrared emitter (max. 3 × 1500 W). The coating speed was adjusted to 5 m/min for the performed trials 1–6 (Table 2). A drying time of 4 s (power output 100%) with infrared (IR) heating and 120 s (power output 100%) with hot air (HA) heating was used. During the simultaneous drying regime (IR + HA), drying was first conducted for four seconds with the IR emitter, followed by 120 s with the HA heater. Due to the shorter drying time and unequal energy input moisture content of dried samples is variable. The IR and HA dried samples might manifest higher amounts of moisture compared to intensive simultaneous drying method (IR + HA). For that reason, all samples dried with different drying methods were equally conditioned in climate chamber for 48 h at 23 °C and 50% RH prior characterization [33].

### 2.3. Design of Coating Trials

Table 2 lists the coating parameters which were altered in the six trials. The first three trials were performed with one layer of chitosan (see Table 2). The second layer was applied under the same conditions with a targeted coat weight of 3 g/m^2^ for one layer and 6 g/m^2^ for two layers. The top side of the paper was coated and all samples were one-side coated. The applied dry coat-weight was controlled for each coated sheet by weighing and only sheets that deviated less than 5% from the target coat-weight were accepted. The wet layer thickness was controlled by the use of a grooved rod and in both cases was 60 µm.

### 2.4. Preparation of Chitosan-Based Coating Solution

**Figure 1:**
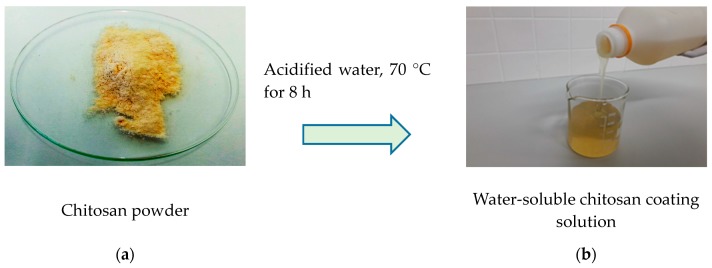
Chitosan powder in its native form (**a**) and chitosan fully dissolved in water, used for surface treatment of the fibre-based substrate (**b**).

The desired solid content of the chitosan formulation used in coating trials was 5% (*w*/*w*). This concentration was achieved by dissolving the chitosan in hot water and adjusting the pH using acetic acid (pure, technical grade from Rotipuran) (see Figure 1). The distilled water was heated to 50 °C and stirred at 600 rpm for ten minutes. Subsequently, the chitosan powder was added in portions (1–2 g) to the heated water (held at 50 °C). After dispersion of the chitosan powder the temperature was increased to 70 °C. Small portions of acidic acids were added (0.5–1 mL) to improve and fully solubilize the chitosan, which occurs at a pH lower than 6 [34,35]. This solution was then stirred for 8 h at 70 °C, once the chitosan particles had fully dissolved. Deprotonated (referring to water-soluble chitosan) chitosan is positively charged and charge was monitored by measuring the zeta potential using a Stabino particle charge mapping analyser (Colloid and Particle Metrix GmbH, Inning am, Ammersee, Germany). The average zeta potential of the chitosan was +475.0 ± 45.3 mV, which indicated that the solution was stable.

### 2.5. Flow-Behaviour of Water-Soluble Chitosan Solution

The viscosity of the 5% (*w*/*w*) chitosan solution was measured using a rotational rheometer (Paar Physica MCR 301, Anton Paar, GmbH, Germany) at low, medium and high shear rates under standard conditions. In the Table 3 mean values and corresponding standard deviations of four-fold viscosity measurements for each shear rate are given (*n* = 4). The shear rates were chosen to represent the conditions in an industrial coating process regarding pumping, mixing and metering in a film press using a rod. Shear rates during pumping and mixing lie in the range of 0.1 to 1000 1/s, whereas in grooved rod metering shear rates of 50,000 1/s and higher are observed. The viscosity of the chitosan decreased at higher shear rates, meaning that the chitosan solution displayed shear-thinning flow behaviour. This is an important characteristic of a coating material intended to serve as a surface-treatment chemical in film press coating, as it is directly related to both the material coatability and to the runability of the coating machine [36,37,38].

### 2.6. Determination of Dry Coat Weight; Basis Weight, Density and Thickness

The uncoated and chitosan-coated paper samples were conditioned according to the standard [33] in a climate room for 48 h at 50% relative humidity ±3% and 23 ± 1 °C. Conditioned samples were used for the assessment of basic physical properties, such as basis weight, thickness, density and dry coat weight as well as the evaluation of mechanical and barrier properties [39,40]. The basis weight of the uncoated raw paper was determined using an analytical balance (Sartorius BP 210D, Sartorius AG, Göttingen, Germany) and the thicknesses of the samples were measured with a Lehmann thickness tester. After determining the basis weight, the dry pick-up weight (equal to the dry coat weight) for coated samples was calculated using Equation (1), where the basis weight of the coated paper (cp) was subtracted from that of the uncoated paper (up).

(1) $$\mathrm{Dry}\;\mathrm{coat}\;\mathrm{weight}\;\left[\frac{g}{m^{2}}\right]=\mathrm{Basis}\;\mathrm{weight}\;\mathrm{cp}\;\left[\frac{g}{m^{2}}\right]-\mathrm{Basis}\;\mathrm{weight}\;\mathrm{up}\;\left[\frac{g}{m^{2}}\right]\;$$

### 2.7. Evaluation of Mechanical Properties

Important tensile properties for packaging materials, such as the tensile strength (TS) and tensile energy absorption (TEA), were measured using a universal tensile tester Zwick Z010 (Zwick, Ulm, Germany) and the data were analysed using the software testXpert V3.0. The measurements were performed for both the cross direction (CD) and machine direction (MD). The test speed (strain rate) of the Zwick tensile tester was the same for all samples (5 mm/min). Samples were cut into strips, each with a width of 15 mm and length of 15 cm. The clamp distance of the Zwick tensile tester at start position was 50 mm for all samples. The determination of the burst pressure was performed using an L & W bursting strength tester SE 180 [41]. The bursting strength of the samples is expressed as burst pressure. The clamp force and gauge pressure of the burst strength tester were 2900 N and 0.22 MPa, respectively.

### 2.8. Determination of Barrier Properties, Wettability and Water Absorption

The water vapor permeability, expressed as water vapor transmission rate (WVTR), was measured using a gravimetric method according to the standard at 23 °C and 50% RH [42]. The test area used was 50 cm^2^ and the measurements were performed over 24 h. Silica gel was used as a desiccant (20 g of silica gel with indicator, Carl Roth GmbH, Karlsruhe, Germany), pre-dried at 160 °C for 24 h. The calculation of the water vapor transmission rate (g/m^2^ 24 h) was performed using Equation (2), where Δm represents mass gains in grams over the time period t, t is the time interval in hours and A is the sample test area exposed to desiccant.

(2) $$\;WVTR=\frac{\Delta m}{A*\Delta t}$$

The grease resistance of the uncoated and coated samples was measured according to Tappi 559 paperboard [43]. This procedure is better known as the KIT test and represents the resistance of a material to penetration by oils and fats. Prior to testing the samples, fresh KIT testing solutions based on different mass ratios of hexane (analytical grade, Merck, Vienna, Austria), toluene (analytical grade, Carl Roth GmbH, Karlsruhe, Germany) and castor oil (pharmaceutical grade, Merck, Vienna, Austria) were prepared according to the standard. In total, twelve different solutions (1–12) were prepared and used for the assessment of grease resistance.

The air permeance of the coated samples was evaluated by measuring the air-flow rate according to the Bendtsen method [44]. The test area was 10 cm^2^ and the applied testing pressure was 1.47 kPa. The air permeance (P) in µm/(Pa·s) was calculated by using the Equation (3) in accordance with the Bendtsen method: where q is the air flow rate in mL/min, A is the tested area of the sample (cm^2^) and p is the testing pressure multiplied by factor k (k = 6).

(3) $$P=\frac{q}{k*A*p}$$

The water absorption of the samples was measured using a Frank PTI Cobb tester [45]. The tested area of the samples was 100 cm^2^. The water absorption of the uncoated and coated samples was measured using deionized water for 60 and 1800 s, respectively. The Cobb_60s_ and Cobb_1800s_ were given as the mass of water that was absorbed by sample (g/m^2^) over the given period of time.

The surface wettability (Contact angle) of all samples were measured with deionized water using a Fibrodat 1100 device (Fibro System AB, Stockholm, Sweden) [46]. Samples were cut into strips (10 cm × 1.5 cm) and placed into the sample holder, then covered with double-sided tape. For each strip, 10 drops of water were placed on the strip and the contact angle was determined. The volume of the dosed water drop was 4 µL, which was kept constant for all samples.

### 2.9. Surface Analysis

The characterization of the surfaces was performed at low acceleration voltages of the electron beam (low voltage scanning electron microscopy, LVSEM, Technologies Inc., Salem, OR, USA) using the high resolution scanning electron microscope Zeiss Sigma 300 VP (Zeiss, Oberkochen, Germany) and the Everhart-Thornley Secondary Electron Detector (ETD, SE2 according to the databar of the micrographs, Oberkochen, Germany). All images were acquired at an acceleration voltage of 0.65 kV (i.e., the landing energy of the electrons is 0.65 keV) at small working distances with a magnification of 500× (horizontal image field width: 228.7 μm). Each of the samples was cut in squares measuring 1.5 cm × 1.5 cm. These squares were mounted on SEM stubs using a conductive double-faced adhesive carbon tape with no further preparation since LVSEM enables imaging of surfaces without coating [47].

### 2.10. Statistical Analysis

All data are given as mean values with the corresponding standard deviations. The number of replicates for each measurements is provided in the description of the figures and tables (*n* = number of replicates). An analysis of the experimental data was performed using the ANOVA function in Microsoft Excel with a confidence interval of *p* < 0.05. In order to establish the statistical differences between mean values, an analysis was performed using the Tukey-Kramer test with a confidence interval of *p* < 0.05.

## 3. Results and Discussion

### 3.1. Physical Characterization of Uncoated and Coated Samples

Table 4 shows the basis weight, bulk density, thickness and coat weights for all samples. The bulk density was calculated by division of the basis weight by thickness. As expected, the bulk density of the chitosan-coated samples increased compared to the uncoated paper with the double coated paper showing the highest densities. The dry coat weights were in the desired range of 3 and 6 g/m^2^ with corresponding standard deviations of less than 5%.

### 3.2. Effects of Drying and Chitosan Layers on Surface Topography

The coating layer quality was assessed using scanning electron microscopy (SEM). Conventional, water-based coating solutions, unlike those containing chitosan, usually have viscosities below 100 mPa·s. The lower viscosity of the coating solution promotes a higher degree of penetration into the substrate, which could be beneficial to the development of the material strength properties [37].

**Figure 2:**
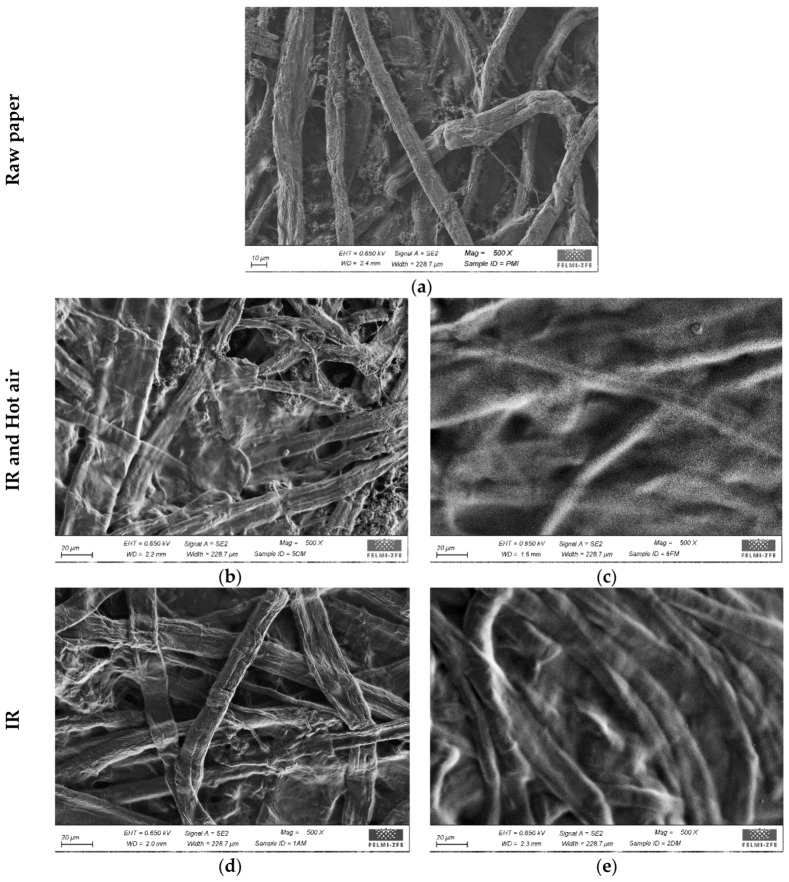

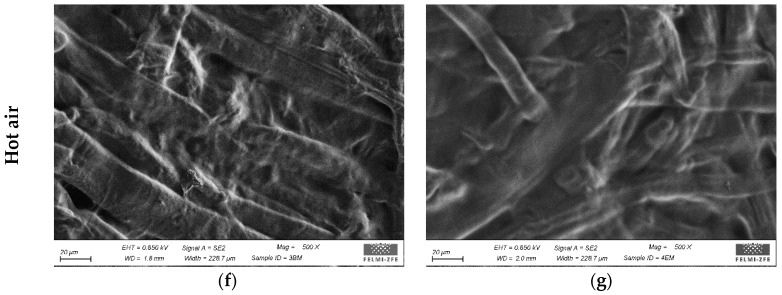
Visualized surface (SEM) features of uncoated raw paper and chitosan-coated samples dried using various methods: (**a**) Raw paper; (**b**) IR + Hot air (1 layer); (**c**) IR + Hot air (2 layers); (**d**) IR (1 layer); (**e**) IR (2 layers); (**f**) Hot air (1 layer); (**g**) Hot air (2 layers).

We used surface visualization to evaluate the differences between uncoated and coated samples and effects of the drying method on the formation of the chitosan layer on the paper surface. In the Figure 2, SEM images with magnifications of 500× are shown for the raw uncoated paper and coated paper samples. Surface of the uncoated raw paper with visible fibres and voids, seems to be very porous, thus enabling chitosan solution to penetrate easily into paper structure. In accordance with SEM images it can be optically seen that after coating of the raw paper, pores and fibres are partially or totally covered with chitosan. Samples coated with one layer show a poorer coating coverage compared to those coated with two layers of chitosan, where a homogenous layer without visible fibres or voids was observed. The differences between paper surface coated with one layer and dried with IR, HA and IR + HA are also observed and might depend on different drying time and drying energy input. We observed that the drying influenced the coverage of the fibres. It is noticeable that the HA and IR + HA drying methods allowed the layer to consolidate more rapidly due to higher drying energy input compared to 100% IR drying method, where the drying was conducted for shorter period of time (see Section 2.2). According to our results, it can be stated that two layers of chitosan are able to close the pores and covers the fibres surface, irrespective of drying method. When it comes to one layer of chitosan, shorter drying leads (IR) to worse surface coverage compared to longer drying (HA and IR + HA).

### 3.3. Effects of Chitosan Coating and Drying Regime on Tensile Strength

**Figure 3:**
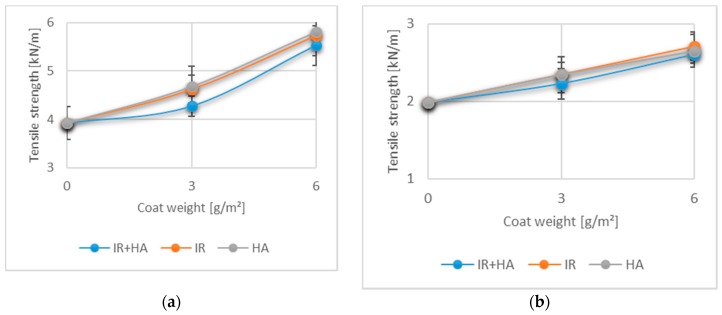
Tensile strength (TS) of paper coated with 3 g/m^2^ (1 layer) and 6 g/m^2^ (2 layers) of chitosan: (**a**) TI for machine direction (MD); (**b**) TI for cross direction (CD). (*n* = 15).

The tensile properties of uncoated fibre-based materials are influenced predominately by the strength and length of individual fibres, bonding strength of fibre-fibre bonds and bonding area [48]. Tensile properties are one of the most important mechanical properties of paper-based packaging materials, due to their direct correlation to material failure during converting operations. Furthermore, they are also an indication of how paper will perform at different loads for some specific packaging application. In Figure 3 the mean values for tensile strength (*n* = 15) with corresponding standard deviations are given.

For both measurements in MD and CD a noticeable increase in the paper tensile strength is observed for the papers coated with one and especially with two chitosan layers. The results of statistical analysis indicated no statistically significant effects of drying on the TS values obtained in both MD and CD for one or two layers (*p* > 0.05). These results agree well with research performed using chitosan films, which found no significant difference between different drying technologies [31]. Furthermore, obtained results are in alignment with published data reporting a positive influence of chitosan coatings on development of fibre-based material strength properties [49,50,51].

### 3.4. Effects of Chitosan and Drying on Tensile Energy Absorption

**Figure 4:**
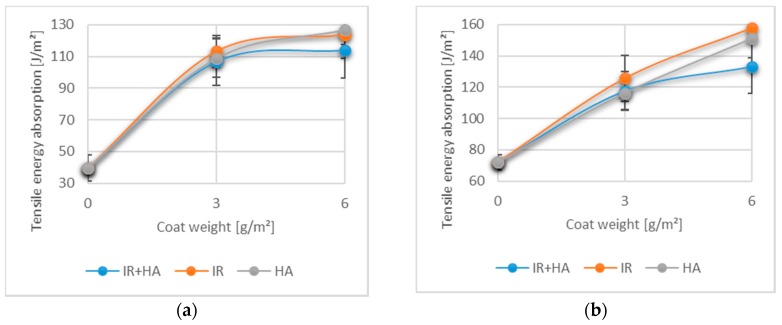
Tensile energy absorption (TEA) of paper coated with 3 g/m^2^ (1 layer) and 6 g/m^2^ (2 layers) of chitosan: (**a**) TEA for machine direction (MD); (**b**) TEA for cross direction (CD). (*n* = 15).

Tensile energy absorption (TEA) is a further significant property of packaging materials and is indicative for the energy taken up by the paper during fracture. It is defined as the total work consumed during tensile testing per unit area of the sample [52,53]. Figure 4 depicts the mean values and corresponding standard deviations for TEA (MD and CD) for one and two chitosan layers. It is evident that, even with one chitosan layer, the TEA in both CD and MD was improved by at least 60% compared to uncoated paper. With two layers of chitosan, an increase in the TEA of at least 85% compared to uncoated paper was observed.

Although the values obtained with IR and HA drying were slightly higher than those obtained with HA + IR, again no statistically significant effects of the drying method were observed for the single or double chitosan coated samples (*p* > 0.05). According to the results of our analysis, chitosan generally has remarkable influence on the tensile energy absorption.

### 3.5. Effects of Chitosan and Drying on Burst Strength

**Figure 5:**
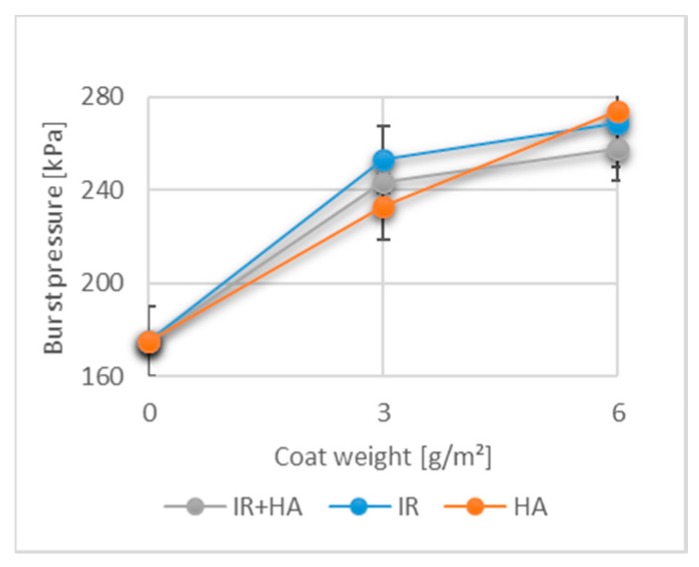
Burst pressure of uncoated paper and paper coated with 3 g/m^2^ (1 layer) and 6 g/m^2^ (2 layers) of chitosan. (*n* = 15).

Together with TI and TEA, bursting strength (see Figure 5) is a further important material property of packaging papers. Although the principle of measurement differs from TI and TEA, it can still be correlated with these values regarding the final application of paper-based packaging materials. Bursting strength is defined as the amount of hydrostatic pressure applied to circular sample area. It provides an indication of the resistance of the paper to rupturing and paper with a low burst strength cannot retain packed goods and tears easily [54]. The burst pressure of raw paper is 175.3 ± 15.0 kPa. By coating the paper with one layer of chitosan, the burst pressure can be enhanced by at least 25%. By adding two layers, the burst strength is improved still further, whereby an increase of 32% compared to that of uncoated paper was achieved.

The interaction between chitosan as a cationic biopolymer and cellulosic fibres seems to be intense, due to fact that the overall paper burst strength was improved. This improvement could be a result of new bonds created between chitosan and the fibres and a greater bonding area created between fibres. The development of the burst pressure follows the same trend that has already been seen with the tensile strength and tensile absorption energy. The mean values obtained from samples coated with one or two layers and then dried using either the IR, HA, or both methods, were not significantly different (*p* > 0.05) meaning that the increase in the burst strength was definitely affected by the higher coat weights and can potentially be improved greatly by using industrially produced chitosan.

### 3.6. Effects of Drying and Chitosan Layers on Air Permeance

**Figure 6:**
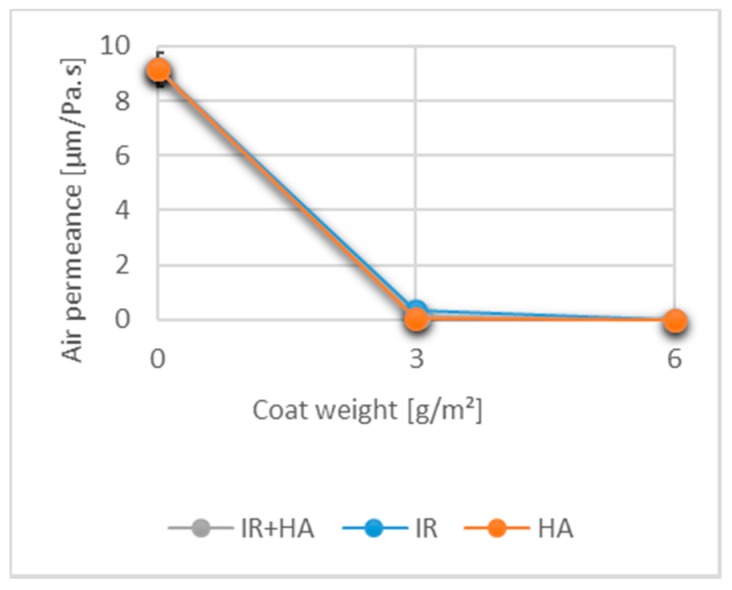
Air permeance for uncoated paper and coated substrates with one and two layers of chitosan. (*n* = 15).

The air permeance of uncoated and coated samples is shown in Figure 6. The air permeance of uncoated paper (9.2 ± 0.6 µm/Pa·s) was significantly higher than that of paper with one chitosan layer regardless of the drying method used. The air permeance values of the single coated samples dried using the IR (0.32 ± 0.01 µm/Pa·s), IR + HA (0.11 ± 0.05 µm/Pa·s) and HA (0.02 ± 0.01 µm/Pa·s) correlated with the observation from the SEM images. For all three drying techniques there was some penetration of the chitosan into the paper structure. This prevented full film formation, yet it resulted in a lower air flow. Statistical evaluation of the results, however, indicated, that the values were not significantly different. Two layers of chitosan, irrespective of the drying method used, reduced the airflow to 0 mL/min which indicates full film formation and surface closure. These results correlate well with the SEM images where a clear film formation was observed when two chitosan layers were applied to the paper making the sample impermeable to air.

### 3.7. Effects of Drying and Chitosan Layers on Greases Resistance

**Figure 7:**
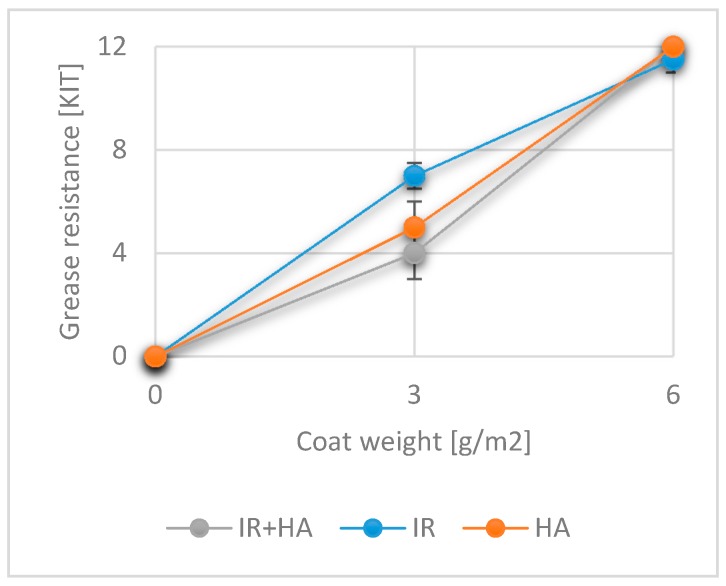
Grease resistance as measured with the KIT test for uncoated substrates and coated substrates with one and two layers of chitosan. (KIT, *n* = 6).

Grease (oil and fats) resistance is an important property of packaging materials. Depending on the future applications, coated material can come into direct contact with different types of oil and fat, which originate from different sources. The KIT test employed to measure the oil and fat resistance of our samples coated with one layer of chitosan gave the following results: 4.0 ± 1.0 (IR + HA), 5.0 ± 1.0 (HA) and 7.0 ± 0.5 (IR) (see Figure 7).

According to the results of the ANOVA analysis, the p-value was equal or greater 0.05, meaning that values are significantly different. Thus, the Tukey-Kramer test was performed to ascertain the differences between the use of the different drying methods. The fat resistance with IR drying was significantly different from that of IR + HA (one sided test), however, no significant difference was observed with HA drying.

Irrespective of the drying method used, the KIT values for samples with two layers reached a maximum of twelve with a corresponding standard deviation of less than 5%. The behaviour of chitosan depends on the pH environment, level of de-acetylation, degree of polymerization and interactions with the substrate. It acts act as a hydrophilic agent which should be beneficial for grease resistance [38,55].

### 3.8. Effects of Drying and Chitosan Layers on the Water Vapor Transmission Rate

**Figure 8:**
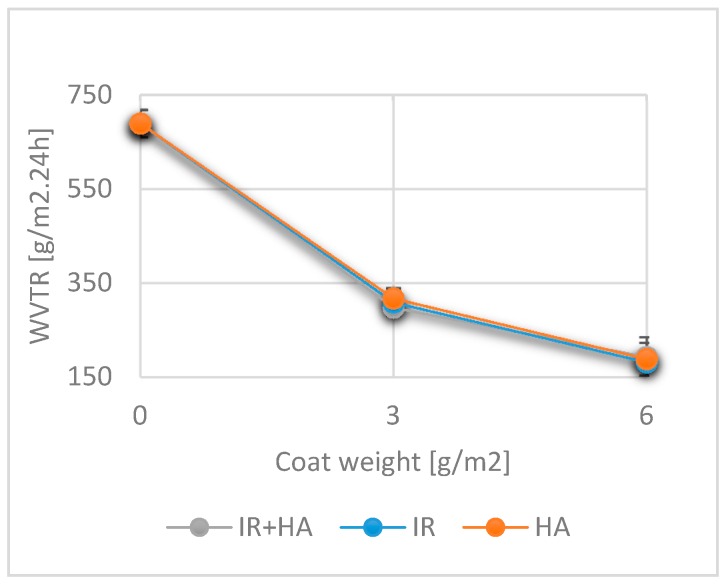
Water vapor transmission rates for uncoated paper and chitosan-coated paper. (*n* = 6).

The water vapor transmission rate (WVTR) is one of the most frequently measured and important properties of food packaging materials. The controlled transmission of water vapor into packaging crucially affects the preservation of packed goods. Moisture can alter the quality of packaged goods by allowing the growth of microorganisms, increasing spoilage rates and shortening the product shelf life. Especially dry food and its quality features (e.g., crispness) are affected by uncontrolled water vapor transmission into packaging [3,4]. The average WVTR values measured for uncoated (689.7 ± 29.2 g/m^2^ 24 h) and coated paper are given in Figure 8. By adding one layer of chitosan, the WVTR was reduced by at least 50% for all drying methods. Adding two layers of chitosan prevented more than 70% of total water vapor transmission compared to the uncoated sample. For both single and double coated samples the effect of the drying regime showed no statistically significant influence.

These results lead us to the conclusion than chitosan, as a polymeric material, is able to impregnate the paper structure, close the pores, repress and reduce the transmission of water vapor. We believe that the largest part of the reduction in water vapor transmission is due to the closing of the pores as demonstrated by the air permeance measurements, Figure 6. However, unlike the air permeance water vapor transmission does not go down to zero at 6 g/m^2^ coat weight. This can be attributed to the hydrophilic nature of the chitosan which enables water vapor diffusion through the closed paper surface. The results obtained are in accordance with those of previously reported investigations [31,56,57].

### 3.9. Effects of Drying and Chitosan Layers on Wettability and Water Absorption

**Figure 9:**
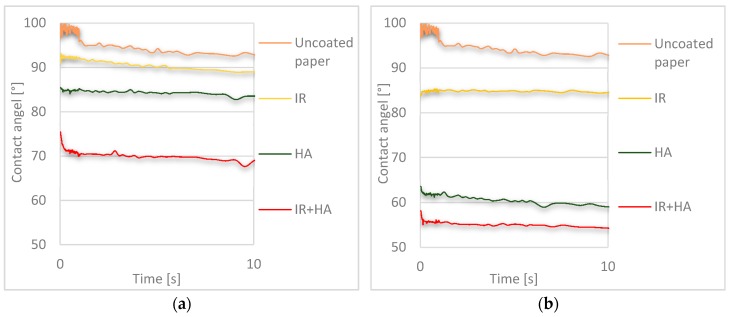
Contact angle of uncoated samples and coated samples measured with deionized water for 10 s: (**a**) Cobb 60 s; (**b**) Cobb 1800 s. (*n* = 10).

**Figure 10:**
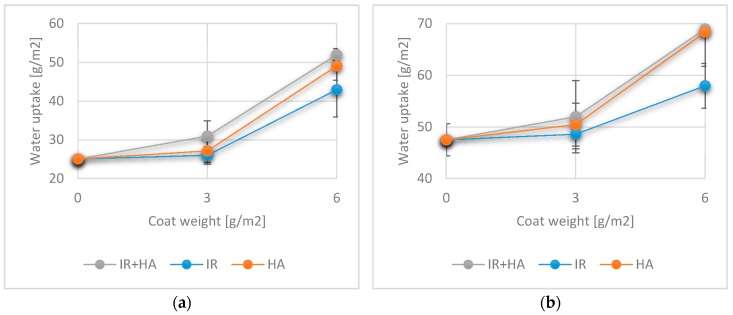
Cobb values for 60 s (**a**) and 1800 s (**b**) with uncoated samples and coated samples with one and two layers of chitosan. (*n* = 8).

The wettability of chitosan-coated and uncoated substrates was studied by measuring the contact angle over ten seconds using deionized water (Figure 9). Water absorption as a material bulk property was determined with Cobb measurements according to ISO 535 for 60 and 1800 s (Figure 10). The uncoated substrate, which was internally sized with an AKD size, had a higher contact angle (~90°) compared to the chitosan-coated samples. When adding one layer of the hydrophilic chitosan, the contact angle was reduced by nearly 5° with the IR, 10° with the HA and around 25° with the HA + IR drying methods. The lower influence for IR drying correlates well to the poorer coverage of the fibres by chitosan as observed in the SEM image (see Figure 2). As a result, the paper surface, which shows a high contact angle due to internal sizing with AKD, is more exposed to water compared to coated papers dried with the HA and IR + HA methods and the contact angle is more similar to the one of the uncoated paper. A similar observation was also made for samples with two chitosan layers, with the reduction in the contact angle being larger due to the higher coat weights. For IR drying the contact angle was reduced by up to 10°, using HA drying by 35–40° and for IR + HA by 40–45°. Based on these measurements, we can state that different drying methods have an effect on the wettability of single and double chitosan coated samples. However, the wettability definitely correlates with a higher amount of the hydrophilic chitosan coated onto the surface.

The water absorption of materials is a time-dependent property and is measured as the capacity of the material to absorb water over a certain amount of time. The Cobb values of the uncoated substrate showed a higher uptake for 1800 s (47.5 ± 3.12 g/m^2^) compared to the uptake for 60 s (25.09 ± 0.48 g/m^2^). Water absorption over 60 s and 1800 s increased for samples coated with one layer of chitosan and further increased, when applying two chitosan layers. This correlation of higher amounts of chitosan coated onto the paper surface to the observed higher values for water absorption agrees with data found in the literature [58].

Comparison of the different drying regimes showed significantly different absorption values at 60 s and 1800 s for IR drying compared to the two other drying methods for both single and double coated papers. These results correlate well with the contact angle measurements, where IR drying also had a different effects on the surface wettability compared to other two drying methods.

According to the results of our investigations, a difference between the surface and bulk properties can be distinguished regarding the influence of chitosan application. The contact angle mostly depends on surface chemistry and presence of pores or voids and it was influenced by altering these parameters using chitosan as a coating material. Different drying methods seemed to affect the consolidation and retention of the chitosan layer on the paper surface, as seen in the SEM images and thus also the penetration of chitosan into the paper structure. For this reason, the wettability was also changed and the hydrophilicity of chitosan contributed to the measurement of lower contact angles and these contributions can be influenced by using different drying methods. On the other hand, bulk properties such as water absorption or grease resistance do not necessarily depend on the surface properties but rather on the material composition. In our case, one layer of 3 g/m^2^ of chitosan seemed to be sufficient to impregnate the paper and to penetrate into the network structure, thus, changing the material composition. By adding two layers, this impregnation was followed by film formation, whereby both the bulk and surface composition were altered. This resulted in moderate changes in bulk properties such as water absorption, when one layer was added and a significant impact when two layers were added, independent of the drying method used.

## 4. Conclusions

Application of chitosan in surface treatment of fibre-based material improved the mechanical properties such as tensile strength, burst strength and tensile energy absorption. Voids and pores of the uncoated paper were partially covered with one layer of chitosan and fully covered with two layers, thus reducing the air or water vapor permeability. Air permeance can be reduced to 0 µm/Pa·s depending on the number of layers added. The water vapor transmission rate was also reduced compared to raw paper, however it due to the hydrophilic character of chitosan it does not go down to zero. The surface of coated paper was hydrophilized by chitosan addition as demonstrated by a lower water contact angle, the water absorption increased. Grease resistance of chitosan-coated paper is moderate with one coating layer (KIT = 4 to 7) and high with two layers (KIT = 12).

Based on the measurements and results of the statistical analyses, the barrier and mechanical properties of coated material are not significantly impacted by the use of the different drying methods. While not significant in statistical tests the mechanical properties of IR + HA drying are worse in nearly every case and the surface hydrophilicity is higher, which indicates a systematic trend. We think that better surface retention of the chitosan due to higher energy input during IR + HA drying causes better film building with this drying method. These differences between the drying techniques could be a future approach to altering the surface functions of chitosan-coated paper.

According to our results it can be also stated that one layer of chitosan, is not sufficient to form the film onto paper surface. Therefore, for some specific application either substrate with smoother structure should be used or at least two chitosan layers must be applied in order to create clear homogenous film onto surface, irrespective of drying method used. The mass percentage of chitosan in the paper coated with 3 g/m^2^ and 6 g/m^2^ was around 4% and 8%, respectively, in terms of the paper basis weight. Considering that the mass percentage of conventional chemicals used as strength or barrier additives in paper-based packaging materials can exceed 10%, chitosan performed excellent as a strength and barrier additive [54]. With respect to the different drying methods, we conclude that chitosan performs well with infrared (IR), hot air (HA), or simultaneously drying (IR + HA) methods and, therefore, represents a potential functional coating material that can be used in an industrial production environment.

## Figures and Tables

**Table 1:** Properties of industrially produced chitosan used for the preparation of the coating formulation [32].

Raw Material	Ash Content [%]	Molecular Weight [kDa]	Degree of Deacetylation [%]	Particle Diameter [µm]	Dynamic Viscosity * [mPa·s]
Chitin from crustaceans	≤1	115	90	≤200	135

* Viscosity of 1% chitosan (*w*/*w*) in 1% (*w*/*w*) acetic acid under standard conditions.

**Table 2:** Design of coating trial with defined layer thickness, number of layers, drying regimes and coat weights. Trials with one layer of chitosan are indicated by trial numbers 1, 2 and 3 (light blue) and trials with two chitosan layers are indicated by 4, 5 and 6 (light green).

Trial	Wet Layer Thickness [µm]	Number of Layers	Drying Regime	Dry Coat Weights [g/m³]
1	60	1	IR + Hot air	3 g/m^2^
2	60	1	IR	
3	60	1	Hot air	
4	60	2	IR + Hot air	6 g/m^2^
5	60	2	IR	
6	60	2	Hot air	

**Table 3:** Viscosity of chitosan, shear rates, corresponding operation stages and units (*n* = 4).

Operation Stage	Operation Units	Shear Rate [1/s]	Viscosity [mPa·s]
1	Pumping	0.1	6760.1 ± 834.4
2	Mixing	1000	762.5 ± 44.6
3	Coating	50 000	59.5 ± 2.6

**Table 4:** Basic characterization: thicknesses, basis weight, bulk densities, dry coat weights and number of chitosan layers of substrate and coated samples from trials 1–6 (*n* = 15).

Specimen	No. of Chitosan Layers	Thickness (µm)	Basis Weight (g·m^−2^)	Bulk Density (g·cm^−3^)	Dry Coat Weight (g·m^−2^)
Substrate	-	95.8 ± 1.2	72.3 ± 0.5	0.75 ± 0.01	0
1 (IR + HA)		98.2 ± 1.0	75.4 ± 0.3	0.77 ± 0.01	3.1 ± 0.1
2 (IR)	1	98.0 ± 1.0	75.3 ± 0.4	0.77 ± 0.02	3.0 ± 0.1
3 (HA)		98.4 ± 1.1	75.4 ± 0.6	0.77 ± 0.01	3.1 ± 0.1
4 (IR + HA)		100.1 ± 1.3	78.3 ± 0.6	0.78 ± 0.01	6.0 ± 0.2
5 (IR)	2	99.9 ± 1.7	78.4 ± 0.7	0.78 ± 0.01	6.1 ± 0.2
6 (HA)		100.0 ± 1.4	78.4 ± 0.4	0.78 ± 0.02	6.1 ± 0.1
